# Transplanted spleen stromal cells with osteogenic potential support ectopic myelopoiesis

**DOI:** 10.1371/journal.pone.0223416

**Published:** 2019-10-04

**Authors:** Helen C. O’Neill, Hong K. Lim, Pravin Periasamy, Lavanya Kumarappan, Jonathan K. H. Tan, Terence J. O’Neill

**Affiliations:** 1 Clem Jones Research Centre for Regenerative Medicine, Bond University, Gold Coast, Queensland, Australia; 2 Research School of Biology, Australian National University, Canberra, Australian Capital Territory, Australia; 3 Department of Microbiology, Yoo Long School of Medicine, National University of Singapore, Singapore, Singapore; 4 Big Data Centre, Bond Business School, Bond University, Gold Coast, Queensland, Australia; Emory University, UNITED STATES

## Abstract

Spleen stromal lines which support *in vitro* hematopoiesis are investigated for their lineage origin and hematopoietic support function *in vivo*. Marker expression and gene profiling identify a lineage relationship with mesenchymal stem cells and perivascular reticular cells described recently in bone marrow. Stromal lines commonly express *Cxcl12*, *Pdgfra* and *Pdgfr* typical of bone marrow derived perivascular reticular cells but reflect a unique cell type in terms of other gene and marker expression. Their classification as osteoprogenitors is confirmed through ability to undergo osteogenic, but not adipogenic or chondrogenic differentiation. Some stromal lines were shown to form ectopic niches for HSCs following engraftment under the kidney capsule of NOD/SCID mice. The presence of myeloid cells and a higher representation of a specific dendritic-like cell type over other myeloid cells within grafts was consistent with previous *in vitro* evidence of hematopoietic support capacity. These studies reinforce the role of perivascular/perisinusoidal reticular cells in hematopoiesis and implicate such cells as niches for hematopoiesis in spleen.

## Introduction

Both mouse and human spleen retain low numbers of long-term resident hematopoietic stem cells (HSCs) [1–4] suggesting that the spleen may play a ‘steady-state’ hematopoietic role. Spleen also supports extramedullary hematopoiesis driven by stress or infection when HSCs mobilize out of bone marrow and into blood and peripheral tissues like spleen, liver and brain [5]. Hematopoiesis in spleen occurs in the sinusoidal-rich red pulp region, supported by evidence that mobilized HSCs entering spleen from bone marrow via blood localize in the red pulp, and that mature myeloid cells are abundant in red pulp [6].

Recent studies have identified PDGFR^+^ perisinusoidal stromal cells in the red pulp region of murine spleen in association with HSCs under conditions of extramedullary hematopoiesis [7]. Mesenchymal progenitor-like cells overexpressing *Hox11* have also been shown to selectively localize in the perifollicular region of red pulp of murine spleen as a source of HSC niche factors [8]. While evidence for HSC niches in spleen is increasing, very little is known about the stromal cells which support hematopoiesis and the hematopoietic cells which are produced, and whether these represent the full spectrum of blood cells, or restricted hematopoietic cell development. Studies from this lab have also identified a role for spleen in the production of a distinct dendritic-like cell type ‘L-DC’ which has been detected in both human and murine spleen [9–11]. These observations reinforce spleen as both an active site for hematopoiesis and as a reservoir for developing myeloid cells.

We have investigated the role of splenic stroma in hematopoiesis since it was first discovered that long-term spleen cultures can support hematopoiesis [12]. Splenic stromal lines overlaid with hematopoietic progenitors, support long-term *in vitro* cultures with production of distinct myeloid cells [13]. Cultures give transient production of several myeloid cell types including cells resembling conventional dendritic cells (cDC), along with continuous production of the distinct subset identified as ‘L-DC’ [14]. Hematopoietic progenitors are maintained in long-term spleen cultures, evident through marker and gene expression studies [15, 16], and through ability to transfer long-term reconstitution upon adoptive transfer of cells into irradiated host mice [17].

A number of splenic stromal lines were isolated from long-term spleen cultures which were subsequently shown to support hematopoiesis in overlaid bone marrow progenitors [18, 19]. The STX3 splenic stromal cell line was derived from a long-term culture of spleen which had lost hematopoietic cells with passage over many years [20]. Multiple clonal lines were established from STX3 [18, 21], and several were shown to support hematopoiesis when overlaid with hematopoietic progenitors derived from fetal liver or bone marrow [13, 19, 22–24]. In particular, STX3, 5G3, 2A8, 10C9 and 7G10 stroma are reliable *in vitro* supporters of hematopoiesis [25] and 5G3 has been used in many studies to date [26, 27]. These cells specifically support the continuous production of L-DC from retained progenitors, and the transient production of other myeloid cells [23, 28]. In contrast, 3B5 is a non-supporter of hematopoiesis *in vitro* [25]. In particular, 5G3 has supports hematopoiesis when overlaid with sorted bone marrow-derived HSCs or multipotential progenitors (MPPs), with production of only L-DCs [24]. These stromal lines can also support common myeloid progenitors (CMPs) or common dendritic progenitors (CDPs), but give only transient production of myeloid cells and no long-term L-DC production [13]. Consistent with these findings is evidence that CD146^+^ perivascular cells from human adipose tissue can maintain HSCs in co-cultures [29].

Splenic stromal lines including STX3, 5G3, 3B5, 7G10, 2A8 and 10C9 are characterized here in terms of their individual lineage origin and similarity with perivascular reticular stromal cell types reported in bone marrow. In terms of lineage origin, the 5G3 supporter line is compared with the 3B5 non-supporter 3B5 line. Stromal lines are also for their ability to form an ectopic niche *in vivo* as a more physiological test of hematopoietic support capacity.

## Materials and methods

### Animals

Specific pathogen-free C57BL/6J (*H-2K*^*b*:^ *CD45*.*2*) mice aged 4 to 8 weeks and NOD/SCID (*H-2K*^*d*^: *CD45*.*1*) mice aged between 6 and 9 weeks, were obtained from the John Curtin School of Medical Research (JCSMR: Canberra, ACT, Australia). Mice were housed and handled according to protocols approved by the Animal Experimentation Ethics Committee at the Australian National University (ANU: Canberra, ACT, Australia). Experimentation followed the guidelines of the *Australian code for the care and use of animals for scientific purposes 8*^*th*^ *Edition (2013)*.

### Stromal cell cultures

The 5G3, 3B5, 7G10, 2A8 and 10C9 stromal lines were cloned from STX3 splenic stroma derived from a long-term culture of spleen from B10.A(2R) mice (*H-2K*^*k*^) [18, 21, 30]. Stromal cell lines were grown from frozen stocks and passaged up to five times by transfer of cells to new flasks following trypsinization using 0.25% trypsin-EDTA treatment to dissociate cells. Stromal cells were cultured at 37°C in 5% CO_2_ in air in Dulbecco’s modified Eagle’s medium (DMEM) supplemented with 10% fetal calf serum, 5x10^-4^M 2-mercaptoethanol, 10mM HEPES, 100U/ml penicillin, 100g/ml streptomycin, 4mg/l glucose, 6mg/l folic acid, 36mg/l L-asparagine and 116mg/l L-asparagine hydrochloric acid (sDMEM).

### Enrichment of myeloid lineage cells and progenitors

Single cell suspensions of spleen were prepared by forcing tissue through a fine wire grid, followed by lysis of red blood cells through exposure to isotonic buffer (140mM NH_4_CL, 17mM Tris Base [pH 7.5]). MACS® magnetic bead technology (Miltenyi Biotec: Gladbach, Germany) was used to prepare T and B cell depleted splenocytes as described previously [28]. Following exposure of spleen leukocytes to biotinylated antibodies specific for CD19 and Thy1.2, cells were passed through LS or MS columns (Miltenyi Biotec) containing anti-biotin MACS® magnetic beads placed in a SuperMACS® magnetic separator (Miltenyi Biotec). Flow-through cells were washed through and collected for experimentation.

### Establishment of co-cultures

The capacity of stromal lines to support hematopoiesis was assessed by overlay of myeloid cells and progenitors above stromal cell monolayers grown to 80–90% confluency followed by co-culture for several weeks [19, 23, 28]. T and B cell depleted splenocytes were prepared and plated at 1–5 x 10^4^ cells/ml in flasks containing stromal cell monolayers. Co-cultures were held at 37°C, 5% CO_2_ in air and 97% humidity. At 7-day intervals, non-adherent cells were collected by gently shaking the flask with removal and replacement of supernatant. Cell yield was determined by counting and cells analyzed for surface marker expression by antibody staining and flow cytometric analysis.

### Light microscopy

Microscopy was used to assess cell morphology. Cells were observed and photographed by bright-field and inverted phase contrast microscopy using a DMIRE2 inverted research microscope (Leica: North Ryde, NSW, Australia) equipped with a DFC digital camera (Leica).

### Immunofluorescence microscopy

Tissue was embedded in Tissue-Tek OCT^TM^ compound (10.24% polyvinyl alcohol, 4.26% polyethylene glycol, 85.50% non-reactive ingredient) and sections of 10μm thickness prepared using a Reichert-Jung 2800 Frigocut cryostat (Reichert-Jung: Depew, New York, USA). Sections were acetone-fixed for 15 minutes at 4°C and air dried for an hour before storage at -80°C. Frozen sections were left to thaw at room temperature before staining. Tissue sections were first blocked with 10% heat inactivated fetal calf serum in PBS for 45 minutes at room temperature and then stained with fluorochrome-labelled antibody for 45 minutes at room temperature. Slides were washed thrice in PBS for 5 minutes. Stained sections were examined using a Leica TCS SP5 Confocal microscope (Leica Microsystems: North Ryde, NSW, Australia). Nuclear DNA was stained with DAPI (Sigma-Aldrich, St Louis, MO, USA). Antibodies used were specific for CD11b (M1/70), CD11c (HL3), H-2K^k^ (36-7-5), CD150 (TC15-12F12.2), CD41 (MWReg30) and CD48 (HM48-1) all purchased from Biolegend (San Diego, CA, USA).

### Flow cytometric analysis

Procedures for staining cells with antibodies for flow cytometric analysis of cell surface marker expression have been described in detail previously [28, 31]. Briefly, cells were suspended in buffer (DMEM with 0.1% sodium azide and 1% FCS) and then incubated with ‘Fc block’ specific for FcγII/IIIR (CD32/CD16) (eBiosciences: San Diego, CA, USA), prior to labeling with multiple fluorochrome-conjugated antibodies used at minimal saturating concentrations. Antibodies used were specific for: CD11c (N418), CD11b (M1/70) and MHC-II (AF6-120.1) from eBiosciences; CD45.2 (104), F4/80 (C1:A3-1), B220 (RA3-6B2), CD3e (145-2C11), Thy1.2 (30H-12), cKit (2B8), CD150 (TC15-12F12.2), CD48 (HM48-1), CD29 (HMß1-1), gp38 (8.1.1), CD140a (APA5), VCAM-1 (429), CD105 (MJ7/18), CD51 (RMV-7), CD54 (YN1/1.7.4), CD31 (390), H-2K^k^ (36-7-5), MHC-II (25-9-17), Flt3 (A2F10), Sca1 (2B8), CD45.1 (A20) from Biolegend. Multicolour flow cytometric analysis was performed on an LSRII flow cytometer (Becton Dickinson: Franklin Lakes, NJ, USA) after addition of 1μg/ml propidium iodide (PI) for flow cytometric discrimination of dead cells. The specificity of antibody binding was monitored through use of isotype matched control antibodies used to set gates to delineate specific antibody binding. Splenic leukocytes were also used as a positive control for binding of antibodies specific to hematopoietic cells.

### Culture of MSC from bone marrow

Cultures enriched for MSCs were established according to published methods [32]. This involved repeated medium change of cultured bone marrow cells followed by trypsinization to provide cells for differentiation studies. To establish cultures, bone marrow was flushed from the femur and tibiae of C57BL/6J mice using DMEM and filtered through a 70μm nylon mesh filter. Red blood cells were removed by lysis with isotonic buffer (140mM NH_4_Cl, 17mM Tris Base, pH7.5), and cells cultured at 10^7^ cells/ml in sDMEM. After 24 hours, non-adherent cells were removed by medium replacement, and cells then maintained by medium exchange every 3 to 4 days. After 14 to 18 days, cells were subcultured following treatment with 0.25% trypsin-EDTA for 2 minutes at 37°C and cells replated at a density of 10^5^ cells/ml in preparation for culture under conditions which stimulate osteogenesis, adipogenesis or chondrogenesis.

### Osteogenic differentiation

Bone marrow cultures established at 10^5^ cells/ml were cultured in a-MEM medium supplemented with 10% fetal calf serum, 5x10^-4^M 2-mercaptoethanol, 10mM HEPES, 100U/ml penicillin and 100ug/ml streptomycin (Sigma-Aldrich). To induce osteogenesis, medium was supplemented with 10^-8^M dexamethasone, 100uM ascorbic acid 2-phosphate and 10mM β-glycerophosphate (all Sigma-Aldrich) [32, 33]. Cultures were maintained for up to 4 weeks. Cells were not passaged over this time but medium was replaced every 3 days. Concurrently, stromal lines were maintained at ~10^5^ cells/ml in the same culture medium by passaging every 4 days throughout the differentiation period using 0.25% trypsin-EDTA to dissociate cells. Parallel cultures of 5G3 and 3B5 were passaged in normal medium at the same density as an undifferentiated control. Cells were harvested at several time points and stored at -80°C for later analysis of alkaline phosphatase activity and gene expression. After 21 and 23 days of culture in osteogenic medium, cells were washed twice with PBS, fixed with 70% ice-cold ethanol for 1 hour at 20°C and stained with 40mM Alizarin Red S (pH 4.1–4.3) for 10 minutes at 20°C [34]. Excess stain was removed with a distilled water wash and cells observed microscopically.

### Adipogenic differentiation

To induce adipogenic differentiation, bone marrow cells were seeded at 10^5^ cells/ml, cultured as monolayers in sDMEM to attain confluency. They were then maintained in differentiation medium composed of sDMEM supplemented with 10^-6^M dexamethasone (Sigma-Aldrich), 10ug/ml insulin (Becton Dickinson, Bedford, MA, USA), 0.45M 3-isobutyl-1-methylxanthine (Sigma-Aldrich) and 0.2mM indomethacin (Sigma-Aldrich) [35–37]. After 3 days, medium was replaced with adipogenic maintenance medium comprising sDMEM supplemented with 10μg/ml insulin for two days. After 14 to 16 days of adipogenic induction, cells were washed twice with PBS, fixed with 10% neutral buffered formalin for 1 hour and stained with fresh Oil Red O solution for 10 minutes to detect accumulation of cytoplasmic triglycerides. Excess stain was removed with several distilled water washes and cells photographed by microscopy. Stain was prepared by mixing three volumes of 0.5% Oil Red O powder (w/v) in isopropanol with two volumes of distilled water. Bone marrow cultures were not passaged, but 5G3 and 3B5 cultures were passaged at medium change as described above for osteogenic differentiation. Parallel cultures of 5G3 and 3B5 maintained in sDMEM medium served as undifferentiated controls.

### Chondrogenic differentiation

To induce chondrogenesis, cultured bone marrow cells were seeded at 10^5^ cells/ml in serum-free sDMEM supplemented with 10^−7^ dexamethasone, 0.35 mM L-proline, 100uM ascorbic acid-2-phosphate (Sigma-Aldrich), ITS+ premix (6.25 ug/ml insulin, 6.25 ug/ml transferrin, 6.25 ng/ml selenious acid, 1.25 mg/ml bovine serum albumin and 5.35 ug/ml linolenic acid) (Becton Dickinson) and 10 ng/ml TGF-β3 (R&D Systems: Minneapolis, MN, USA). Medium was changed every 3 days with addition of TGF-ß3 and maintained for a period of 3 weeks [35, 36]. Bone marrow cultures were not passaged, but 5G3 and 3B5 cultures were passaged at medium change as described above for osteogenic differentiation. Parallel cultures of 5G3 and 3B5 were maintained in sDMEM medium as undifferentiated controls. Cells were subjected to Alcian Blue staining to confirm chondrogenic differentiation after 21 to 23 days. Cells were washed twice with PBS, fixed with 10% neutral buffered formalin for 1 hour at 20°C and stained with 1% Alcian Blue solution (pH 2.5) for 20 minutes. Excess stain was removed with several washes of distilled water and cells photographed microscopically to detect accumulation of sulphated proteoglycans within the extracellular matrix of chondrocytes.

### Alkaline phosphatase assay

To determine the expression of alkaline phosphatase by cells undergoing osteogenesis, the SensoLyte alkaline phosphatase assay kit (Anaspec: Freemont, CA, USA) was used according to the manufacturer’s instructions. Cell lysates from cultures undergoing induced osteogenesis were prepared at days 0, 4, 8, 12, 16 and 20 days of culture and mixed with the p-nitrophenyl phosphate substrate with incubation at 37°C. The optical density of p-nitrophenol phosphate was determined spectrophotometrically at 405nm. Alkaline phosphatase activity was calculated as μmoles/min/ng protein.

### Quantitative RT-PCR

Cells cultured under osteogenic conditions were collected at 0, 8, 16 and 24 days. RNA was extracted using the RNeasy mini kit (Qiagen: SABiosciences: Valencia, CA, USA) as per the manufacturer’s instructions. The concentration and purity of RNA was determined spectroscopically. RNA was converted to cDNA using the RT^2^ First Strand Synthesis kit (Qiagen) as per the manufacturer’s instructions. The resultant cDNA was then used as a template for amplification by Real Time PCR. Primers used were purchased from SABioscience (Frederick, MD, USA) specific for genes: *Actb* (beta-actin: PPM02945A), *Bglap* (bone gamma carboxyglutamate protein/osteocalcin: PPM04465E), *Alpl* (Alkaline phosphatase: PPM03155A), *Spp1* (secreted phosphoprotein 1/osteopontin: PPM03648C), *Ibsp*, (integrin binding sialoprotein/bone sialoprotein: PPM35999A), and *Sp7* (Sp7 transcription factor 7/osterix: PPM35999A). The cDNA primers and RT^2^ SYBR Green Master mix were added to RNA in a ratio of 1:6.25:5.25, respectively, and samples plated in a 384 well plate and loaded on to a Light Cycler 480 (Roche: Castle Hill, NSW, Australia). Cycling conditions used were: 1 cycle for 10 minutes at 95°C to activate DNA Taq Polymerase, followed by 45 cycles of 15 sec at 95°C for extension and 1 minute at 60°C to quantitate PCR products. Data analysis was performed initially using Roche Light Cycler 480 software v1.2.9.11. B-actin was used a house-keeping control gene for normalization. The absolute Quantification (2^nd^ derivative max) method at high confidence was used to obtain a C_T_ value or threshold cycle. C_T_ values were exported into Microsoft Excel and the relative gene expression levels estimated using the 2^-ΔΔCT^ method, taking gene expression at day 0 as reference.

### Transcriptome analysis

Total RNA was isolated from stromal cell lines using the RNeasy mini kit and following the manufacturer’s protocol (Qiagen), and the concentration and purity of RNA determined spectrophotometrically. Double stranded cDNA was synthesized in a two-step process. The first strand of cDNA was synthesised using T7-(dT)_24_ primers and Superscript II reverse transcriptase (Invitrogen Life Technologies: Mount Waverley, VIC, Australia). This was followed by second strand cDNA synthesis. Double-stranded DNA was then purified using phenol-chloroform together with phase-lock gels (Brinkmann Instruments: Westbury, NY, USA). Subsequent procedures were performed by the Biomolecular Resources Facility (JCSMR, ANU). *In vitro* transcription and biotin labeling were performed using the BioArray High Yield RNA Transcript Labelling Kit (Affymetrix). cRNA was cleaned up on RNeasy Spin columns (Qiagen), fragmented, and then labeled with biotin. Fragmented and labelled cRNA was hybridized to Murine Genome 430v2 genechips following the manufacturer’s procedures (Affymetrix: Santa Clara, CA, USA). These were washed and stained on the fluidics station (Affymetrix) ahead of scanning and image analysis using a Gene Array Scanner (Affymetrix). Comparative analysis across two experiments (Expt 1: STX3, 2A8, 5G3, 3B5; Expt 2: 7G10, 5G3, 3B5) involved standardization of genechips and removal of variation due to batch effect. Scanned images of genechips were processed using Microarray Suite 5.0 software (MAS5.0; Affymetrix).

### Analysis of microarray data

Gene expression data was analysed using Partek to give average signal values and p values by Stephen Ohms (Biomolecular Resource Facility: ANU, Canberra, Australia). Microsoft Excel files containing all information on probeset numbers, genes, signal values and p-values were prepared for Principal Components Analysis. Data mining on the basis of average signal values was used to determine gene expression associated with known functions or lineages. Heatmap analysis and agglomerative hierarchial clustering using the Lance-Williams dissimilarity formula involved the use of R project (http://www/r-project.org/).

### Ectopic grafting

Two methods for graft preparation were employed. Due to the overall difficulty of the procedure, outcomes of both procedures were taken as informative.

By the collagen sponge method: *in vitro* grown stromal cells were harvested by trypsinization and filtered through a 70μm cell strainer to produce a single cell suspension. To prepare each graft, ~1–2 x 10^6^ cells were sedimented (1200 rpm, 4°C, 5 minutes) and resuspended in 20ul sDMEM using a P20 pipette tip sealed at the opening with Parafilm (1200 rpm, 4°C, 10 minutes). The cell pellet formed was then released on to a collagen sponge construct soaked in 400ul sDMEM and placed in one well of a 6-well plate for overnight incubation.

By the Matrigel method: *in vitro* grown stromal cells were harvested and filtered as described above. Cells were then pelleted and resuspended in 10-20uL Matrigel (Matrigel Matrix, standard formulation 8–12 mg/mL: Corning, New York, USA). The Matrigel-cell mix was withdrawn into a 1-10uL Wiretol capillary micropipette (Drummond, Broomall, PA, USA) and allowed to soldify at room temperature before injection.

Prior to grafting, NOD/SCID mice were anaesthetized with 3.5% isofluorane in oxygen. A 20-25mm skin incision was made in the region of the left kidney, the kidney was exteriorized and a 2-4mm incision made in the capsule. Fine-tipped forceps were used to create a pocket between the capsule and the underlying parenchyma into which the graft was implanted. Upon completion, the kidney was replaced back into the body cavity and sutures and skin staples were used to close the incision wounds. The graft was dissected out from the renal capsule at 1, 3 or 4 weeks post-grafting, and cut into smaller pieces before treatment with collagenase to dissociate cells for analysis using antibody staining and flow cytometry. In some cases multiple separated grafts were applied to the same kidney to increase the chance of engraftment. In these animals, individual grafts were analysed independently.

### Statistical analysis

Data are presented as mean ± standard error (SE) for sample size n. The Wilcoxon Rank Sum test was used to assess significance (p ≤ 0.05).

## Results

### Splenic stroma which support in vitro hematopoiesis reflect perivascular reticular cells

**Fig 1:**
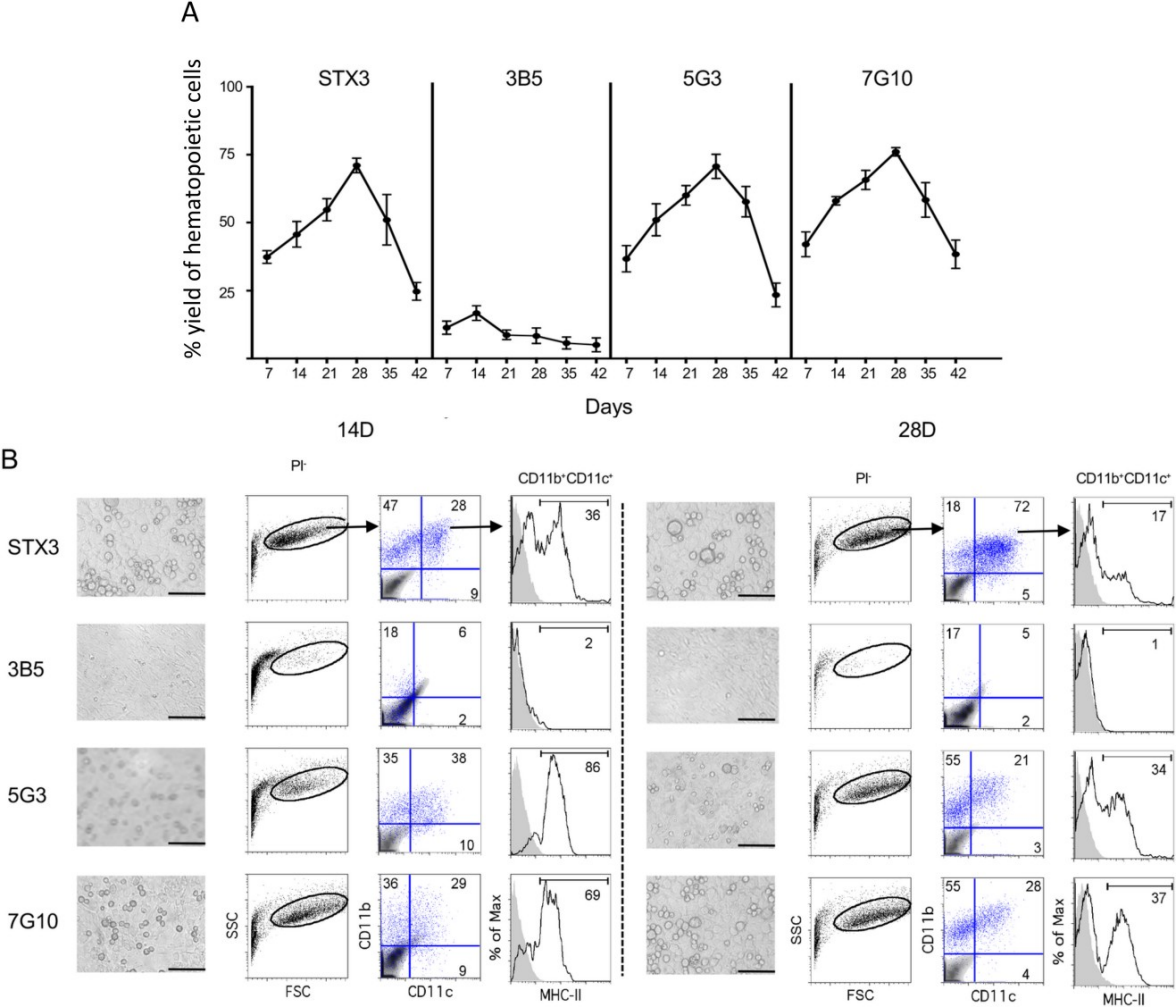
Stromal co-cultures support hematopoiesis. Co-cultures were established in triplicate by overlay of T and B cell depleted splenocytes at 0.5–1 x 10^5^ cells/ml over near-confluent stroma. Controls comprised stroma alone and ‘T/B depleted splenocytes’. (A) Co-cultures were established with 3B5, 5G3, 7G10 and STX3 stroma. Non-adherent cells were collected at 7-day intervals for estimation of percent (%) live cell recovery relative to input cell number using trypan blue exclusion for cell counting. Data represent mean ± standard error (n = 3). Cell production by 3B5 compared with all other stroma was statistically significant at each time point (p≤0.05). (B) Co-cultures were established over 5G3 stroma. Cell production was compared and photographed at 14 and 28 days under brightfield (Objective 20x, bar 250μm). Non-adherent cells were collected and stained with antibodies specific for CD11c, CD11b and MHC-II, or with isotype controls to set gates. Propidium iodide (PI) staining prior to flow cytometry allowed gating of PI^-^ (live) cells and FSC and SSC analysis was used to gate large (FSC^hi^) cells for subsequent analysis. Numbers shown in quadrants represent percent positive cells. Culture of stroma alone have no cell production (not shown).

In line with their splenic origin, stromal lines STX3, 5G3, 3B5, 7G10 and 2A8 (not shown) support myelopoiesis for an extended period when overlaid with splenocytes depleted of lymphoid and erythroid cells. Fig 1A shows production of non-adherent cells out to 42 days, with 3B5 the poorest supporter. Co-cultures reveal loosely adherent cells over a confluent stromal monolayer (Fig 1B). Three main cell types are produced out to 14 days: CD11b^+^CD11c^-^ (myeloid cells), CD11b^+^CD11c^+^MHC-II^+^ (cDC-like cells) and CD11b^+^CD11c^+^MHC-II^-^ (L-DC), with the latter dominating out to 28 days (Fig 1B). The population of cDC-like cells has recently been characterized as regulatory DC [38].This pattern of restricted hematopoiesis with long-term production of the novel dendritic-like cell ‘L-DC’ has been demonstrated consistently over time using bone marrow as a source of progenitors [19, 23, 39].

Further studies concentrated on 5G3 and 3B5, since these stromal lines were the best *in vitro* growers making experimentation more feasible. Phenotypic analysis of the 5G3 and 3B5 stroma was used initially to determine relationship with endothelial, mesenchymal and perivascular reticular cells, otherwise called CXCL12-abundant reticular (CAR) cells. Confluent stroma was dissociated and stained with a range of antibodies. These cells showed no expression of markers of the hematopoietic lineage (Table 1). Stroma also lacked markers of mature endothelial cells like CD31 and CD54. They did express markers common to mesenchymal stem cell (MSCs) including CD105, CD29, CD106 (VCAM1), LY6A (Sca1) and CD90 (Thy1.2) [40–43]. Stroma reflect CAR cells with expression of markers like CD106, LY6A and CD90 [44–48]. 3B5 and 5G3 are also reflective of stromal cells reported to support hematopoiesis in that they express CD51 and CD140A [44, 49]. Their resemblance with perivascular reticular cells described in mouse, and CAR cells described in humans, was evident through expression of CD51, VCAM1 and PDGFRA (CD140a) [44, 49, 50]. 5G3 and 3B5 also expressed podoplanin (gp38), previously described as a marker of fibroblastic reticular cells in spleen [51].

**Table 1:** Cell surface markers expressed by stroma.

Cell surface marker	Cell type specificity ^a^^,^^b^	MFI - 5G3 ^c^	MFI - 3B5 ^c^
**Stromal cells**			
gp38	FRC	6720 ± 839	4110± 814
CD105	MSC, CA, RPS	493 ± 80.9	464 ± 140
CD29	MSC, FRC	2070 ± 243	2520 ± 450
CD90 (Thy1.2)	MSC, CAR cells	6730 ± 457	2640 ± 446
CD51	CAR cells	294 ± 51.0	299 ± 94.5
LY6A (Sca-1)	MSC, CAR cells	3160 ± 579	279 ± 83.3
CD106 (VCAM1)	MSC, CAR cells, FRC, MRC	448 ± 47.3	179 ± 29.5
CD140a (PDGFRA)	CAR cells, CA, RPS	403 ± 160	123 ± 48.9
CD31	EC	13	13
CD54 (ICAM-1)	EC	9	7
**Hematopoietic cells** ^**d**^			
CD3e	T cell	0	0
B220	B cell	2	4
CD150	Hematopoietic cells	2	0
CD48	Hematopoietic cells	6	1
CD11b	Myeloid cells	1	0
MHC II	Antigen presenting cells	71	28
F4/80	Macrophages	0	1
CD11c	Dendritic cells	0	2
Ly6G	Neutrophils	5	6
CD45.2	Hematopoietic cells	36	36

^a^ CAR cells, CA, central arteriole; CXCL12-abundant reticular cells; EC, endothelial cells; FRC, fibroblastic reticular cells; MRC, marginal zone reticular cells; MSC, mesenchymal stem cells; RPS, red pulp sinusoids.

^b^ Mueller and Germain 2009 [43]; Nagasawa et al. 2011 [42]; Oh and Kwon 2010 [40]; Omatsu et al. 2010 [36]; Pinho et al, 2013 [41].

^c^ Mean fluorescence intensity (MFI), shown as mean ± SE (n = 4) for expressed markers.

^d^ Splenic leukocytes were stained as a positive control for hematopoietic markers. Stainings were not duplicated due to the use of overlapping lineage markers.

### Analysis of stroma through gene expression analysis

**Fig 2:**
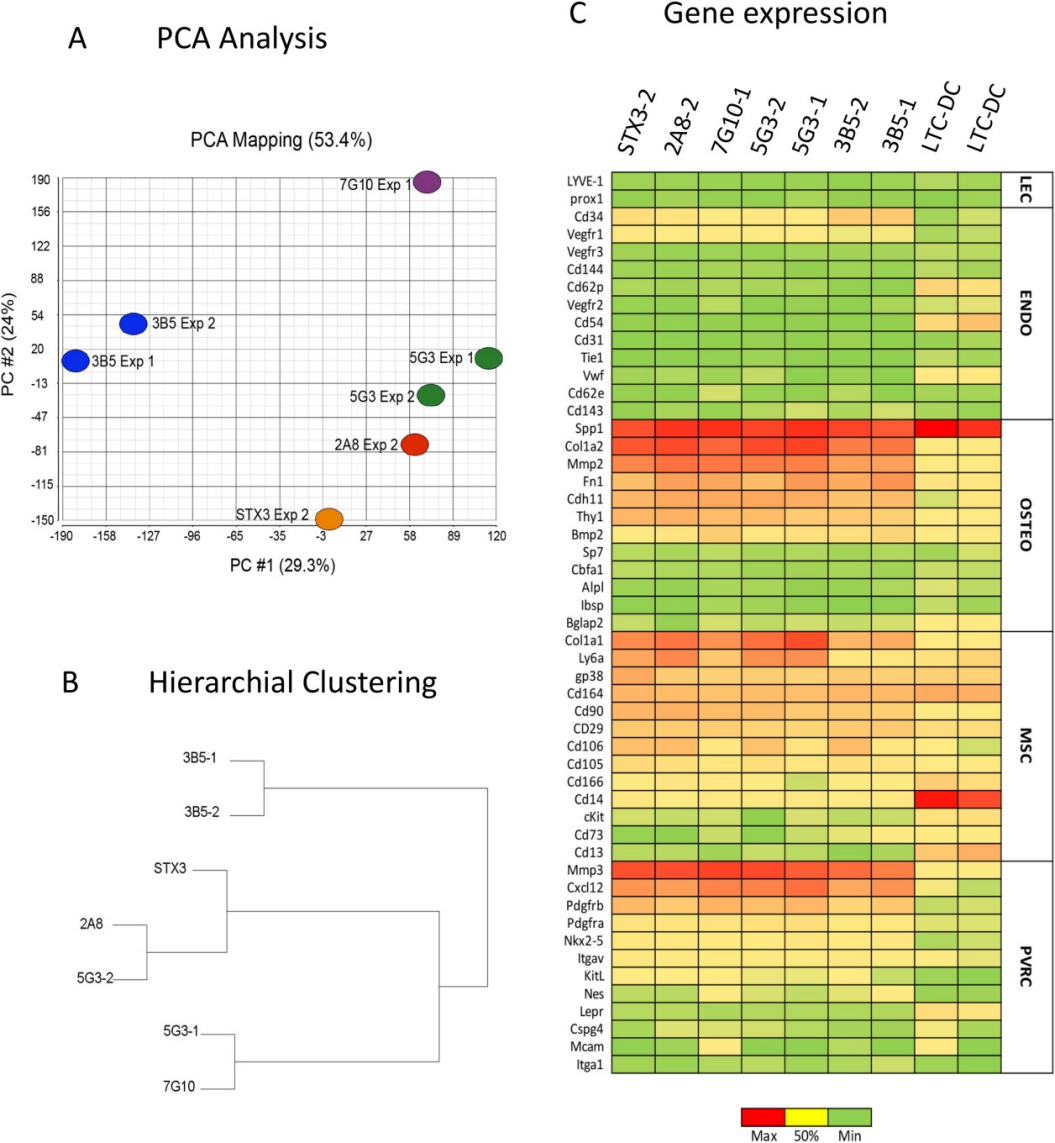
Gene profiling identifies splenic stromal lines as mesenchymal cells. Transcriptome analysis using Affymetrix Murine Genome 430v2 genechips was performed on multiple stromal cell lines in duplicate experiments. Genechips from two experiments were standardized and compared with control hematopoietic cells collected from long-term spleen cultures (LTC-1, LTC-2). (A) Principal Component Analysis (PCA) was used to determine variability in gene expression for each sample. Shown are first (PC#1) and second (PC#2) principal components. (B) Hierarchial clustering was used to analyze the relationship between stromal lines on the basis of gene expression. The dendrogram displays distance between subsets based on clustering 5077 genes selected for analysis on the basis of mean signal value > 1000 for any one stromal line. (C) Data mining was used to plot signal value for genes identified as specific for lymphatic endothelial cells (LEC), endothelial cells (ENDO), osteogenic cells (OSTEO), mesenchymal stem cells (MSC) and perivascular reticular cells (PVRC).

In order to investigate the lineage origin of stromal lines, transcriptome analysis was performed using Affymetrix Murine Genome 430v2 genechips. Two separate experiments were performed comparing multiple stromal lines. Controls included hematopoietic cells isolated from long-term stroma-dependent cultures of spleen which produce myeloid and dendritic-like cells (LTC-DCs). The first analysis looked at the relationship between the stromal cell lines under the hypothesis that 3B5 is distinct from other lines in terms of lineage origin since 3B5 cells are superior *in vitro* supporters of hematopoiesis. Only small differences in overall gene expression between the stromal lines were indicated through Principal Component Analysis (PCA). Greatest variability in gene expression was seen for the first and second principal components, where greatest variability was encountered (29.3% and 24%, respectively) (Fig 2A). 3B5 was distinguished from other stromal lines in the first principal component. Hierarchical clustering was used to identify the relationship between stromal lines on the basis of gene expression. This also confirmed some distinction between 3B5 and all other stroma (Fig 2B). Small differences between the same cell line analysed in different experiments are most likely due to natural variation developing in cell lines following *in vitro* culture. Since all lines derived from the long-term cultured line STX3, it is expected that the variation seen between cloned lines is not due to intrinsic differences between distinct stromal. Despite their value as a model for study, long-term cultured cell lines will not accurately reflect *in vivo* biology.

Data mining was used to investigate the expression of genes reflecting distinct stromal cell types. This involved retrieval of signal values for sets of genes related to the lineage origin and function of MSCs, osteoblastic cells, endothelial cells including lymphatic endothelial cells and perivascular reticular cells. The gene sets used were collated from the literature with lineage-specific genes selected where possible [52–57]. Fig 2C shows a heat map detailing signal value for selected genes. Very similar gene expression was obtained for each of the 5G3, 2A8, 3B5 and STX3 stroma, suggesting a common lineage origin. Hematopoietic cells produced in LTC (LTC-DCs) served as controls, showing distinct gene expression.

None of the stromal lines expressed genes encoding markers specific for endothelial cells like the *Vegfr* family, *Cd144 (*VE-Cadherin), *Cd62p* (P-selectin), *Cd54* (ICAM-1), *Cd31* (PECAM-1), *Tie1* (angiopoietin receptor), *Vwf* (von Willebrand Factor), *Cd62e* (E-selectin), or *Cd143* (Ace) (Fig 2C). 3B5 did weakly express *Cd34*, which encodes a marker of hematopoietic progenitors and MSCs It is also expressed by vascular endothelial cells [58] (Fig 2C) and by a subset of pericytes [59]. *Cd34* expression however is thought to vary with culture and so may not be a reliable marker of lineage [60]. None of the lines expressed the most common marker of mature lymphatic endothelial cells *Lyve-1*, nor the *Prox1* transcription factor [61]. All stromal lines expressed genes related to osteogenesis including *Spp1* (osteopontin) required for osteoblast differentiation, *Col1a2* (bone specific collagen) and *Mmp2* (matrix metalloproteinase 2) involved in bone and cartilage formation (Fig 2C). Stroma also expressed *Fn1* (fibronectin), *Cdh11* (cadherin 11) and *Bmp2* (bone morphogenetic protein 2), all indicative of osteogenic development. Stroma did not express transcription factors essential for osteoblast differentiation including *Cbfa1 and Sp7* (osterix), nor did they express genes reflecting mature osteoblasts like *Alpl* (alkaline phosphatase), *Bglap2* (osteocalcin) and *Ibsp* (bone sialoprotein). Stroma expressed *Col1a1* (collagen type1 alpha 1), *Sca-1* (Ly6A), *Cd164* (sialomucin), *Cd90* (THY1), *Cd29* (integrin b1), *Cd106* (VCAM1) and low levels of *Cd105* (endoglin), all reflecting a relationship with mesenchymal stem/progenitor cells and confirming data shown in Table 1. The stromal lines did not however express *Cd166*, *Cd14*, *cKit*, *Cd73 and Cd13*, reported as markers of mesenchymal cells [62]. The absence of *KitL* (SCF) expression distinguishes these stroma from endothelial cells, and subsets of perivascular reticular cells identified in bone marrow which produce both CXCL12 and SCF [63], but aligns them with cells in spleen which have been identified as HSC niches elements [7]. However, all cell lines expressed *gp38*, expressed by mesenchymal stromal cells in spleen [51].

Stroma also expressed genes consistent with described perivascular reticular subsets found in bone marrow and spleen including high expression of *Cxcl12* and low expression of *KitL* (SCF) which encode known regulators of hematopoiesis [63]. Expression of genes like *Mmp3*, *Cd106*, *Cd51* and *Pdgfrb* are also characteristic of CAR cells [53] and are expressed by each of the stromal cell lines. *Pdgfra* expression was lower than *Pdgfrb* expression, with cell surface marker expression (CD140a) also detectable in Table 1. Expression of *Nkx2-5* was consistent with the mesenchymal lineage origin of cells [64]. Stromal lines were however distinct from bone marrow perivascular reticular subsets which express *Nes*, *Mcam*, *Ng2*, *Lepr* and *Nte5* [65]. In summary, 5G3, 3B5, 2A8, 7G10 and STX3 all show characteristics of mesenchymal, osteogenic cells resembling perivascular reticular cells, with no evidence for expression of genes reflective of endothelial or hematopoietic cells. Stromal cells were clearly distinct from myeloid cells produced in LTC (LTC-DC) which strongly express *Spp1*, *Cd164* and *Cd14* [12] (Fig 2C).

Further evidence of the similarity between the stromal lines comes from common cytokine and chemokine gene expression. Supplementary Fig 1E shows Log_2_ signal values attained by transcriptome analysis for chemokine and cytokine pathway genes selected by SASBiosciences for their PCR arrays. Overall expression appears to be constant across the stromal lines but with a few clear differences. All cell lines expressed very high levels of *Ccl8*, *Mmp2*, *Cxcl12*, *Ccl2*, *Ccl7*, *Cxcl5*, *Cxcl10*, *Csf1*, *Cxcl1* and *Ccl5*, reflective of common functionality in hematopoiesis (S1 Fig). CXCL12 expression is common to perivascular reticular cells in bone marrow, while CSF1 is a myeloid differentiation factor. Notable are the higher expression of *IL-6* and *Cxcr7* by 3B5 over all other lines. IL-6 is an important regulator of inflammation, while CXCR7 is a chemokine receptor which binds CXCL12. Since CXCL12 binding to CXCR4 on HSC is an important regulator of HSC migration and hematopoiesis [45], CXCR7/CXCL12 binding could negatively impact the potential of 3B5 to support *in vitro* hematopoiesis, and this has been reported previously for a stromal cell line MS-5 overexpressing CXCR7 [66]. The stromal cell lines did not express many cytokine genes, although *Mif* was most highly expressed. 3B5 did noticeably upregulate *Lif* and showed downregulation of *Il-16* and *Il-7* compared with other stroma (S1 Fig). LIF (leukemia inhibitory factor) inhibits differentiation of stem cells and could be an important factor in the inability of 3B5 to support *in vitro* hematopoiesis.

### Splenic stromal lines have osteogenic potential

**Fig 3:**
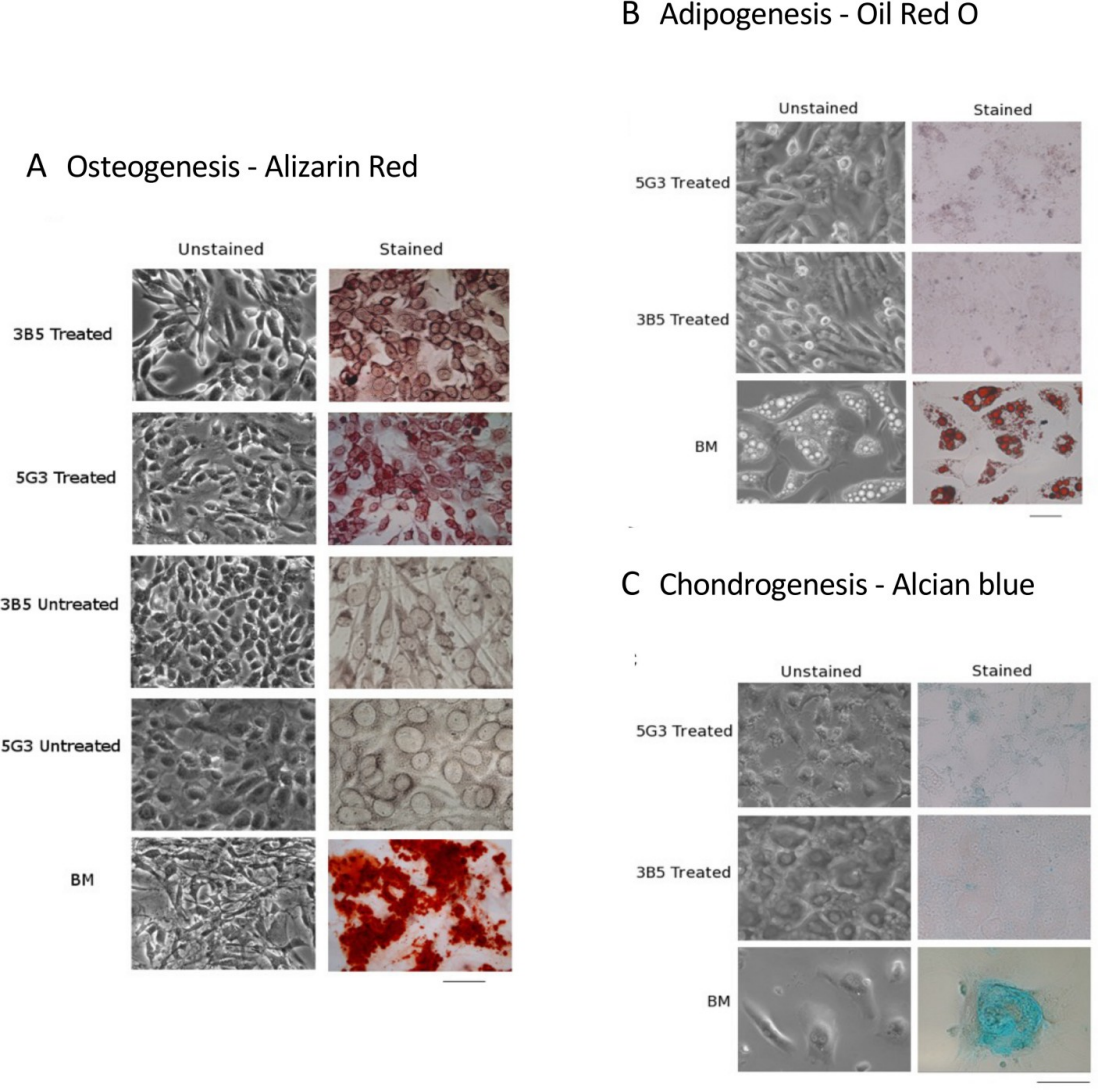
Splenic stromal lines reflect osteoprogenitors. The 5G3 and 3B5 stromal cell lines were cultured under different conditions to induce osteogenic, adipogenic and chondrogenic differentiation, with medium replacement every 3 days. Controls were cultured under normal conditions. In parallel, bone marrow derived MSCs were induced to differentiate under the same growth regime. (A) Cells were cultured for 21 days under mineralization conditions to induce osteogenesis, and then stained after 21 days with Alizarin Red S stain. (B) Cells were cultured for 21 days under chondrogenic conditions and then stained with Alcian Blue. (C) Cells were cultured for 14 days under adipogenic conditions and then stained with Oil Red O. Cells were photographed under bright field microscopy. Unstained cells were photographed under phase. Bar represents 100μm. Data reflect a representative result from replicated experiments.

As a further test of the mesenchymal stem/progenitor phenotype of stromal lines, attempts were made to induce differentiation of 5G3 and 3B5 stroma towards osteogenesis, adipogenesis and chondrogenesis using defined culture conditions. MSCs cultured out of bone marrow were used as controls. By necessity, stromal lines had to be passaged regularly over the experiment to reduce numbers of cells. To measure osteogenesis, cells were cultured under mineralization conditions for a period of 21 days, and then stained with Alizarin Red S to detect osteogenesis based on calcium deposits within the mineralized extracellular matrix [36]. Treated 5G3 and 3B5 stromal lines stained positively after 21 days in comparison with untreated stroma, although staining was moderate in relation to cultured bone marrow cells (Fig 3A).

Stromal cell lines were also cultured under adipogenic conditions, and Oil Red O stain used to assess differentiation after 14 days (Fig 3B). Neither 5G3 nor 3B5 cells showed staining, while cultured bone marrow MSCs showed multiple intracellular lipid droplets accumulating Oil Red O. By 14 days, cultured bone marrow cells developed a mature adipocyte morphology, while no morphological changes were observed in 3B5 or 5G3 cells. Capacity to undergo chondrogenesis was assessed through production of chondrocytes which aggregate and mature, producing extracellular matrix containing aggregated proteoglycans which stain with Alcian Blue. Under these conditions, 5G3 and 3B5 showed no significant change in morphology out to 21 days, and no specific staining with Alcian Blue (Fig 3C). In control cultures of MSC, sulphated mucopolysaccharides characteristic of the extracellular matrix of chondroblasts accumulated Alcian Blue stain, indicating mature cells (Fig 3C). Controls showed round cell morphology and formed small cell aggregates after 14 days. 5G3 showed some extracellular fibrous material across the cell monolayer, perhaps indicative of a low level of chondrogenesis. While 5G3 and 3B5 cells were grown as monolayers, there was no evidence of cellular aggregation although cells became more flattened and acquired a more cuboidal phenotype (Fig 3C).

**Fig 4:**
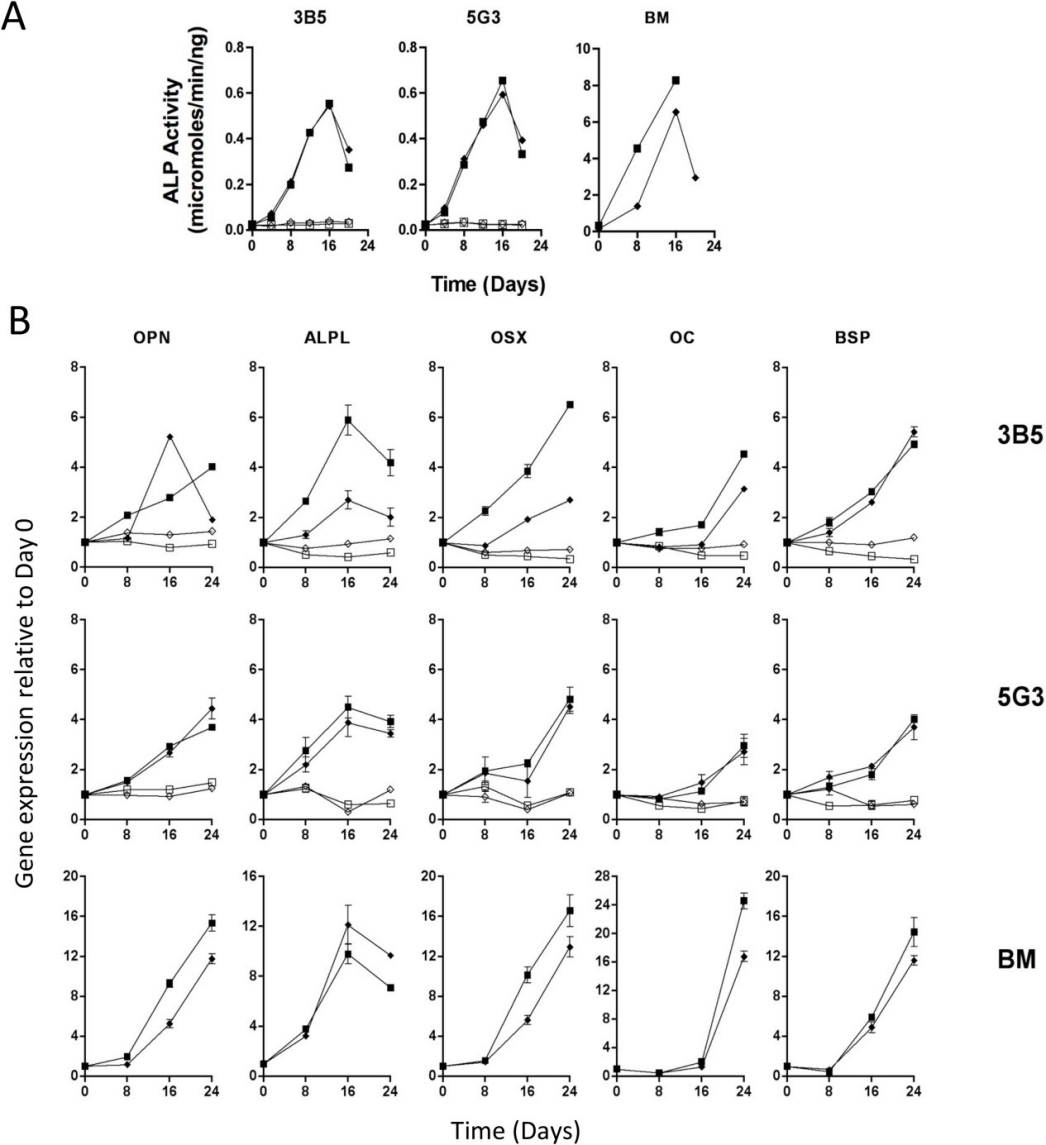
Stromal cells reflect osteogenic progenitors. The 5G3 and 3B5 stromal cell lines were cultured under osteogenic conditions (closed symbols), and under normal growth conditions as a control (open symbols). Duplicate experiments are shown by different symbols, and data points represent mean ° SE of three experimental replicates. Comparison was made with bone marrow-derived mesenchymal cells cultured in parallel under osteogenic conditions. (A) Alkaline phosphatase production (expressed as micromoles/min/ng) was assessed over time using the SensoLyte alkaline phosphatase assay. Day 8, 16 and 24 levels are significantly different from Day 0 values for all samples cultures under osteogenic conditions (p≤0.05). (B) Quantitative RT-PCR was used to measure change in gene expression over time for genes encoding alkaline phosphatase (ALPL), osterix (OSX), osteocalcin (OC), bone sialoprotein (BSP) and osteopontin (OPN). Data are shown as fold change in gene expression at 8-day intervals relative to Day 0 gene expression. For samples cultured under osteogenic conditions, gene expression was significantly different (p≤0.05) from Day 0 levels by Day 16, except for the osteocalcin (OC) by Day 24. Untreated samples showed no significant increase above Day 0 levels.

Alkaline phosphatase production is an early marker of osteoblast differentiation [67, 68]. Enzyme activity was detected in supernatants of 5G3, 3B5 and bone marrow derived MSCs cultured under mineralization conditions using the SensoLyte assay. 5G3 and 3B5 cultured in normal medium served as controls. Alkaline phosphatase was first detected after 4 days in all cultures and increased until 16 days (Fig 4A). Levels detected in bone marrow cultures were up to 10-fold higher than for stromal lines. For cells grown under osteogenic conditions, these values were significantly higher than Day 0 levels after Day 8 (p≤0.05).

To further confirm that the 5G3 and 3B5 stromal lines differentiate to give mature osteoblastic cells, quantitative RT-PCR was used to measure expression of genes related to osteogenesis across 24-day cultures. Genes monitored included *Alpl* which encodes alkaline phosphatase, *Spp1* which encodes osteopontin and *Sp7* encoding osterix as markers of early osteogenic differentiation, with *Bglap2* encoding osteocalcin and *Ibsp* encoding bone sialoprotein as late markers of mineralization associated with osteoblast formation [67, 69]. Over 24 days of culture under mineralization conditions, the expression of genes of interest in cultured bone marrow derived MSCs increased 12- to 24-fold, and by 4- to 6-fold above base levels for 5G3 and 3B5 (Fig 4B). For most genes, expression reached peak levels by 16 or 24 days of culture consistent with changes detected in bone marrow MSCs. Significant increases in gene expression were detected by Day 16 for all genes, but by Day 24 for *Bglap2* encoding Osteocalcin (OC) (p≤0.05) (Fig 4B). Untreated samples showed no significant change above Day 0 levels across the 24 day culture. These changes involved genes reflecting both early and late osteogenesis, although changes were smaller than for bone marrow derived MSCs (Fig 4B). Alkaline phosphatase mRNA expression reached a peak at Day 16 of culture in agreement with enzyme activity measured in parallel experiments (Fig 4). Data from a repeat experiment is shown in S2 Fig.

### Splenic stroma forms ectopic niches which support hematopoiesis

Splenic stromal cell lines studied here were then tested for capacity to form ectopic hematopoietic niches *in vivo* following transplantation into the subcapsular region of adult NOD/SCID mouse kidney. Initial grafting involved the 3B5 and 5G3 lines, with later testing of 10C9, STX3 and 7G10 stromal lines. The different lines showed variable success in graft uptake. Grafting with 3B5 had an overall 70% success rate, but 5G3 gave only 9.1% success rate (S1 Table). Further experiments showed no success for STX3 and 7G10, with 60% success rate for 10C9. These data suggest variable *in vivo* growth capacity amongst the lines. These differences did not relate to time of cell lines in culture since lines were maintained as frozen stocks established after only 4 or 5 passages and were grown for only 4 to 5 passages ahead of each experiment. Since these are continuous cell lines of similar stromal cells, variability could relate to variability in the transformation process individual to each line.

**Fig 5:**
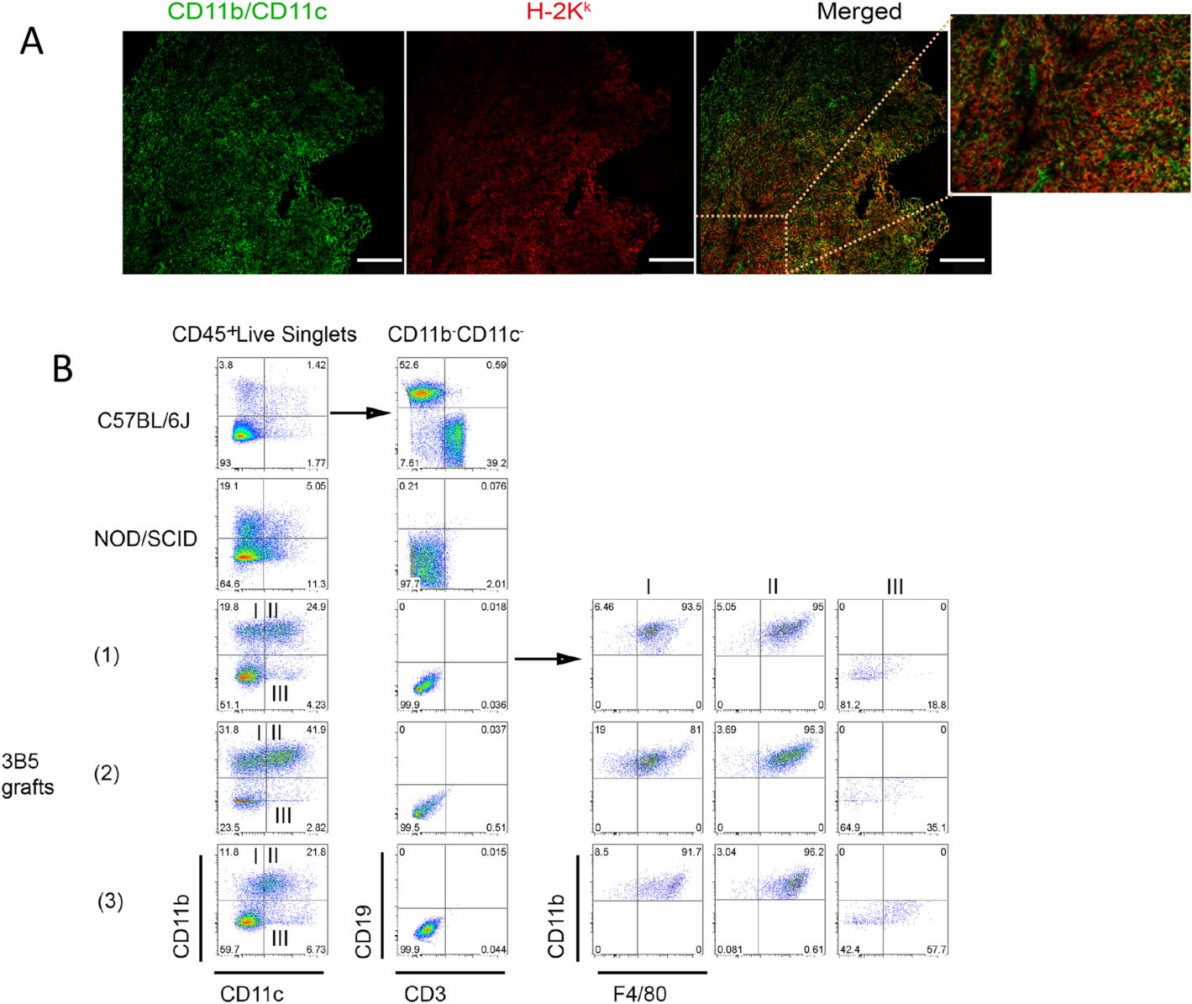
Development of ectopic hematopoietic tissue through grafting of 3B5 stroma. 3B5 stromal cells were grown on a collagen sponge ahead of transplantation under the kidney capsule of NOD/SCID (CD45.1) mice. Grafts were dissected out after 4 weeks and cells dissociated for antibody staining and flow cytometry. (A) Myeloid cells were localized in grafts through staining frozen sections using antibodies specific for CD11b and CD11c along with H-2K^k^ which is specific for donor and not host type cells. Bar represents 25um. (B) Live singlets were gated and staining with CD11b, CD11c and the F4/80 marker was used to identify myeloid subsets. Staining for CD3 and CD19 expression on the gated CD11b^-^CD11c^-^ population was used to identify lymphoid cells. Three grafts from individual mice were analysed and cell composition compared with spleen leukocytes from adult C57BL/6J and NOD/SCID mice.

Grafting 3B5 stroma was very successful, particularly using the collagen sponge method, and section staining at 4 weeks identified prolific stromal cell growth based on staining for MHC-CI (H-2K^k^) (Fig 5A). Successful grafts also contained mature host type myeloid and/or dendritic cells detected by staining for CD11b and CD11c. Due to the small number of cells recovered from grafts, antibodies used to stain myeloid cells were specific for only CD11c, CD11b and F4/80, which is the minimum cocktail needed to distinguish L-DC from monocytes/macrophages and cDC subsets [10]. Flow cytometry identified very consistent myeloid cell types amongst the hematopoietic (CD45^+^) population present in 3B5 grafts (Fig 5B). Many cells in the graft were mature CD11b^+^ and/or CD11c^+^ myeloid cells, with a major population of CD11b^-^CD11c^-^cells reflecting hematopoietic progenitors/precursors. The CD11b^-^CD11c^-^ population contained no CD3^+^ or CD19^+^ cells reflective of T and B lymphocytes in line with a NOD/SCID host origin, showing no infiltration of grafts by mature lymphocytes. Data for myeloid subset representation were collected across multiple grafts (n = 9) (Table 2 and Fig 5B). 3B5 stromal grafts support the presence of a majority (34.4%) population of L-DC with a CD11b^+^CD11c^lo^F4/80^+^ phenotype. The next highest population comprised cells reflective of monocytes/macrophages as CD11b^+^CD11c^-^F4/80^+^ cells (12.5%). A minor 3% population of CD11b^-^CD11c^+^F4/80^-^ plasmacytoid (p)DCs was identified, along with a 1.4% population of CD8^+^ cDCs as CD11b^-^CD11c^+^F4/80^+^ cells, and a 3.2% population of CD11b^+^CD11c^hi^F4/80^+^ CD8^-^ cDCs. No B cells (CD19^+^) or T cells (CD3^+^) were detected as expected for NOD/SCID mice as hosts (Table 2). As with 3B5 grafts, 10C9 grafts comprised a majority population of CD11b^+^CD11c^lo^F4/80^+^ cells reflecting L-DC (S3 Fig). The 3B5 and 10C9 grafts showed almost no evidence of CD11b^+^CD11c^+^F4/80^-^ cells (Fig 5B), a population which would contain eosinophils and neutrophils [10]. This result is consistent with the non-inflammatory environment of these grafts. The presence of many hematopoietic precursors is also consistent with the graft representing a supportive environment for the steady-state development of myeloid cells.

**Table 2:** Representation of hematopoietic cells within 3B5 grafts.

Subset identified ^a^	Predicted cell type	% cells within graft ^b^
CD11b^-^CD11c^-^F4/80^-^**CD3**^**+**^CD19^-^	T cell	0.02 ± 0.01
CD11b^-^CD11c^-^F4/80^-^CD3^-^**CD19**^**+**^	B cell	0.00 ± 0.00
**CD11b**^**+**^**CD11c**^**lo**^**F4/80**^**+**^CD3^-^CD19^-^	L-DC	34.41 ± 8.14
**CD11b**^**+**^CD11c^-^**F4/80**^**+**^CD3^-^CD19^-^	Monocyte/macrophage	12.45 ± 4.15
CD11b^-^**CD11c**^**+**^F4/80^-^CD3^**—**^CD19^-^	Plasmacytoid DC	2.96 ± 0.98
CD11b^-^**CD11c**^**+**^**F4/80**^**+**^CD3^**-**^CD19^-^	CD8^+^ cDC	1.36 ± 0.45
**CD11b**^**+**^**CD11c**^**hi**^**F4/80**^**+**^CD3^-^CD19^-^	CD8^-^ cDC	3.21 ± 1.07

^a^ 3B5 stromal cells were grown on a collagen sponge and transplanted under the kidney capsule of NOD/SCID (CD45.1) mice. Grafts were dissected out after 4 weeks and cells dissociated for analysis by antibody staining and flow cytometry. Subsets were identified by expressed markers only. The unidentified cells in the graft reflect hematopoietic precursors and non-hematopoietic (stromal) cells as verified in Fig 5.

^b^ Data reflect mean ° SE (n = 9). Analysis involved individual grafted mice.

Grafting 5G3 stroma was much less successful than 3B5, contrasting with the superior ability of 5G3 over 3B5 to support *in vitro* hematopoiesis seen in Fig 1A. This finding also contrasts with the excellent *in vitro* growth capacity of both stromal lines. The cell composition of each of three separate grafts of 5G3 made into one mouse is shown in S4 Fig. Grafts clearly contain a small population of CD11b^+^ cells, possibly reflecting two subsets. The absence of CD11b^-^CD11c^-^ cells reflecting early lymphocytes and myeloid precursors was notable in comparison with 3B5 (Fig 5) and 10C9 grafts (S3 Fig). Some CD11b^+^ cells expressed F4/80 suggesting the presence of either L-DCs or cDCs, but others were F4/80^-^ consistent with the presence of neutrophils or eosinophils and reflective of an inflammatory environment. Overall, the cell composition of 5G3 grafts was unusual compared with grafts comprising 3B5 and 10C9 stroma, in that CD11b^-^CD11c^-^ precursor cells comprised only 3–13% of cells in the graft, compared with ~60% for 3B5 and 10C9 grafts. This distinct composition was also consistent with the low success rate of grafting for 5G3 stroma.

### Ectopic stromal grafts maintain HSCs

**Fig 6:**
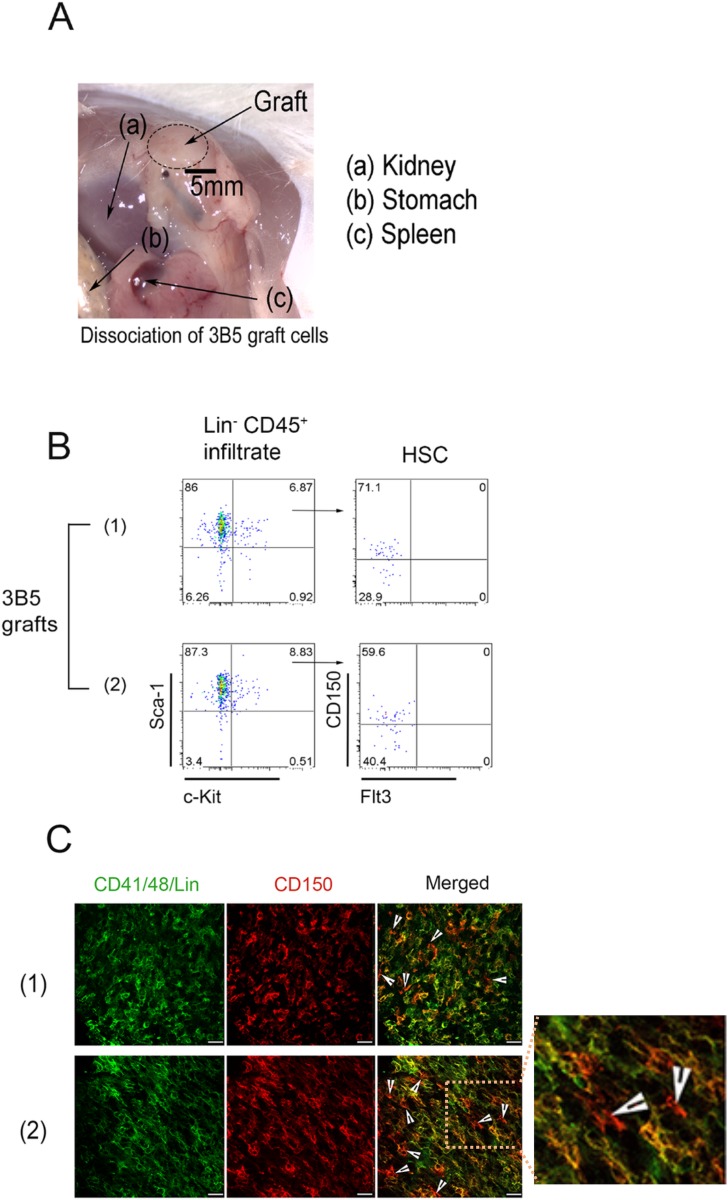
Detection of hematopoietic stem/progenitor cells in ectopic grafts. (A) Grafts of 3B5 stroma were prepared as described in Fig 5. (B) Grafts were dissociated and analysed flow cytometrically to distinguish hematopoietic cells of NOD/SCID host origin (CD45.1). Non-hematopoietic (including 3B5 stromal cells) were gated or excluded from analysis. Hematopoietic stem/progenitor cells were then identified as the lineage-negative (Lin^-^) subset. Further staining for stem cell markers cKit, Sca1, CD150 and Flt3 was used to identify HSCs in the graft as Lin^-^cKit^+^Sca1^+^CD150^+^Flt3^-^ cells. (C) Frozen sections from two separate grafts were stained for CD150 (red) and Lin/CD41/CD48 (green). Arrows identify HSPCs as Lin^-^CD41/48^-^CD150^+^ cells. Bar represents 25um.

An important question is whether mature myeloid and dendritic cells detected in grafts develop *in situ* from colonizing progenitors, or from mature cells migrating into the grafts. This was addressed by immunohistochemical and flow cytometric staining to detect hematopoietic stem/progenitor cells colonizing 3B5 grafts. A successful graft of 3B5 stroma under the kidney capsule is shown in Fig 6A. Tissue fragments were isolated, and collagenase treated for flow cytometric analysis. Staining identified HSCs as a Lin^-^Sca1^+^cKit^+^CD150^+^Flt3^-^ subset of long-term reconstituting HSCs [70] amongst the dissociated graft cells (Fig 6B). This suggested that HSCs had infiltrated two distinct 3B5 grafts on two different animals and could be detected through antibody staining. Section staining also identified hematopoietic stem/progenitor cells as Lin^-^CD150^+^CD41^-^CD48^-^ cells [6] scattered throughout the grafts, along with some mature cells expressing Lin and/or CD41/48 markers (Fig 6C).

## Discussion

Splenic stromal cell lines described here reflect mesenchymal perivascular and perisinusoidal reticular cells previously characterized as a heterogeneous population in bone marrow expressing PDGFRA/B, SCF and CXCL12 [71]. Stromal subset isolation and analysis reflects a difficult study, so that the availability of cell lines represents a rare opportunity to study stromal cell characteristics and function. Evidence presented here also supports a role for splenic stromal cells in formation of an ectopic *in vivo* niche for hematopoiesis giving rise to specific subsets of myeloid cells. This finding has clear clinical importance in terms of expansion of hematopoietic niches outside of the bone environment.

This study was initiated to investigate the lineage origin of stromal cell lines which were known supporters and non-supporters of *in vitro* hematopoiesis. A main question was whether supporters and non-supporters reflected different stromal subsets or even different cell lineages. However, all stromal lines studied were shown to have very similar gene expression profiles reflecting their lineage origin as mesenchymal, osteogenic perivascular cells. PCA analysis confirmed small differences between cell lines, particularly separating 3B5 non-supporters from other supporter lines. In the main, these differences would relate to variation due to genetic and developmental changes following long-term *in vitro* culture than to any differences in cell lineage origin. For 3B5 and 5G3, this is confirmed in that both cell lines retain capacity to differentiate to give osteogenic cells but did not undergo adipogenesis or chondrogenesis.

Gene expression changes over the course of mineralization cultures indicate that 5G3 and 3B5 represent cell line models of osteoprogenitors derived from spleen. Cells of the osteoblastic lineage have long been known to be important regulators of hematopoiesis, but most of this evidence relates to hematopoiesis in bone marrow, and the important role of osteoblasts on the endosteum as a niche for primitive HSCs [48]. That hematopoiesis in bone marrow is dependent on osteoprogenitors has been shown in various conditional deletion murine models such as deletion of *Cxcl12* in osterix-expressing osteoprogenitors which increased HSCs exit from quiescence and entry into cell cycle, followed by mobilization into blood and spleen [72]. To some extent it is an enigma to find osteoprogenitors in spleen, although there is now increasing evidence that perivascular reticular cells in multiple organs show osteoprogenitor characteristics. This raises the question of whether osteogenic potential, and some level of osteogenesis, is a feature of perivascular and perisinusoidal reticular cells developing in spleen, and consistent with their potential to act as niches for HSCs.

Most work to date involving splenic stroma has involved *in vitro* analyses, so that evidence for capacity to produce an ectopic niche *in vivo* is a very new finding which gives promise of translational studies going forward. In order to optimize transplantation outcome, two procedures were used, and both were successful. Cells were either cultured briefly above collagen sponges, or mixed with Matrigel, an extracellular matrix comprising laminin, enactin, and collagen IV [73–75]. Earlier studies involving neonatal spleen capsule grafts showed enlargement of lymphoid follicles for up to 4 weeks post-transplantation [76]. Consistent with that study, 4 weeks post-transplantation was used as a reference point for graft examination and analysis, although analyses at 1 and 3 weeks were also performed to assess outcomes. Previously, Tan et al [76, 77] showed capacity to engraft murine spleen fragments and capsular tissue under the kidney capsule with full development of splenic architecture and inclusion of hematopoietic cells. Those studies also showed that dissociated stromal cells of 3-day spleen enriched for a single marker, either CD31, CD105, CD201 or MadCAM-1, grew to form a successful graft. While those studies led to regeneration of spleen architecture in grafts with formation of red and white pulp regions filled with erythrocytes, lymphocytes and myeloid cells, the grafts formed here with stromal cell lines reflecting perivascular cells do not lead to complete tissue reformation. They do however function appropriately as a niche and support the uptake of HSCs and the development of myeloid cell types of limited range reflecting the myeloid and dendritic-like cells which are produced when stromal lines support restricted hematopoiesis *in vitro*.

Prior to engraftment of cell lines, multiple grafting studies were attempted in this lab involving freshly sorted splenic stromal subsets sorted on the basis of multiple markers. These experiments were very challenging due to the low number of stromal cells which were isolated. So far, these studies have largely been unsuccessful. Our conclusion from these studies is that stromal subsets isolated from spleen on the basis of multiple markers appear to reflect mature cells which do not replicate well *in vivo*. Only less well-defined fractions are likely to contain progenitors or spleen organizer cells which can lead to tissue regeneration. Grafting of stromal cell lines here has been more successful, perhaps due to the transformed and proliferative nature of cultured cell lines. The high success achieved with 3B5 stroma over other stromal lines could relate to a more immature phenotype, and capacity to differentiate on engraftment.

The finding that 3B5 (and 10C9) stromal grafts became infiltrated with hematopoietic cells with clear evidence of CD11b^+^CD11c^lo^F4/80^+^ L-DC-like cells (Figs 5 and S3) contrasts with evidence that 3B5 stroma is a weak supporter of myelopoiesis *in vitro* [19]. Here we show that 3B5 stroma functions as an *in vivo* niche which supports myelopoiesis leading to a range of mature myeloid cells. Indeed, it would appear that these cells develop from bone marrow-derived HSCs which infiltrate the graft, are maintained within the graft, and then differentiate *in situ*. It is also likely that HSCs preferentially differentiate to give L-DC when influenced by the microenvironment created by 3B5 stroma as was demonstrated *in vitro* [27], and that other bone marrow precursors may colonize grafts for their further development. Preferential differentiation of HSCs into L-DCs could account for the high proportion of L-DCs over other monocytes/macrophages and DC subsets within the graft (Table 2).

Numerous *in vitro* studies involving co-cultures of splenic stromal lines with bone marrow-derived hematopoietic stem/progenitor cells have identified production of the L-DC subset which is phenotypically and functionally distinct from all known DC and myeloid subsets described [13, 17, 24, 28]. The physiological relevance of the L-DC subset is supported by evidence for an *in vivo* equivalent subset in murine and human spleen which is not found to be present in other tissue sites [10, 11, 78]. Recent investigation now shows L-DCs to be distinguishable both phenotypically and functionally from all splenic cDC and plasmacytoid (p)DC subsets, and from residential and inflammatory monocytes [10]. We have also shown that L-DC production in *Booreana* (*c-Myb*^*E308*^) mutant mice occurs independently of *c-Myb*, a gene known to regulate hematopoiesis from bone marrow-derived progenitors [79], consistent with L-DC development directly from HSCs present in spleen without formation of a myeloid progenitor. This is consistent with the previously published evidence that L-DCs develop directly from HSCs and MPPs in stromal co-cultures *in vitro* [13, 24]. The hypothesis that L-DCs derive from yolk sac-derived HSC laid down during development of spleen as reported for other myeloid cells [80] can also be considered. There is a precedent for this proposal since tissue-specific antigen presenting cells have been described in sites like skin and brain [81, 82]. L-DCs are not only spleen-specific but also unique as antigen presenting cells, in that they activate only CD8^+^ and not CD4^+^ T cells [83].

Spleen plays a major role in myelopoiesis and it has long been known to support the maturation of myeloid precursors which traffick from bone marrow, the development of cDC from their precursors and the maturation and mobilization of monocytes in response to tissue injury. Now evidence is presented here that spleen contains stromal cell types which can function as a niche for hematopoietic stem and progenitor cells supporting the restricted development of several myeloid cell types. The production ‘L-DC’, and of cDC-like DC resembling regulatory DC, has already been reported *in vitro* and is now confirmed in an *in vivo* model. This study identifies spleen as a competent site to support myelopoiesis and may act as a reservoir for hematopoietic stem and progenitor cells.

The findings presented here have direct relevance to the clinical importance of spleen as an alternative site for hematopoiesis, particularly with loss of bone marrow niches for HSC which occurs with ageing and disease. Spleen is a highly regenerative organ and the stromal cell types which regenerate full tissue development include both endothelial and mesenchymal cell subsets [77, 84]. This study specifically identifies a mesenchymal cell type, which if proliferating, will form ectopic tissue which directs hematopoiesis and specifically myelopoiesis. This study therefore identifies the minimal needs for production of artificial niches *in vivo*. The translational relevance of these findings relates to the ability to harness spleen as an alternate or ectopic site for hematopoiesis so opening new opportunity for therapy in patients with bone marrow disease, or other disease leading to immune system failure.

## Supporting information

S1 Fig Expression of pathway specific genes by stromal lines. Data mining was applied to Affymetrix datasets collected for stromal cells. Log_2_ average signal value was plotted as a heat map, and overlaid dashed blue line represents signal changes around mean). Sets of genes reflecting chemokine, and cytokine pathways represent genes utilized by SASBioscience for their PCR arrays.
(PDF)

S2 Fig Osteogenesis in stromal lines marked by gene expression. A repeat experiment was performed to assess expression of genes reflecting osteogenesis upon culture of 5G3 and 3B5 stroma under mineralization conditions. Quantitative RT-PCR was used to measure change in gene expression over time in culture for genes encoding alkaline phosphatase (ALPL), osterix (OSX), osteocalcin (OC), bone sialoprotein (BSP) and osteopontin (OPN) by 5G3 and 3B5 stromal cells induced to undergo osteogenic differentiation (closed symbols). 5G3 and 3B5 grown under normal culture conditions served as control cells (open symbols). Data are shown as fold change in gene expression at 8-day intervals relative to Day 0 gene expression. Data points represent mean ° SE of three experimental replicates.
(PDF)

S3 Fig Identification of hematopoietic cells in 10C9 ectopic stromal grafts. 10C9 stromal cells were grown on a collagen sponge ahead of transplantation under the kidney capsule of NOD/SCID (CD45.1) mice. Grafts were dissected out after 4 weeks and cells dissociated for antibody staining and flow cytometry. Live singlets were gated and staining for CD11b, CD11c and F4/80 used to identify myeloid subsets. Staining for CD3 and CD19 expression on the gated CD11b^-^CD11c^-^ population was used to identify lymphoid cells. Three separate grafts from individual mice were analysed and cell composition compared with spleen leukocytes from adult C57BL/6J and NOD/SCID mice.
(PDF)

S4 Fig Limited hematopoietic tissue growth with 5G3 stroma grafting. 5G3 stromal cells were grown on a collagen sponge ahead of transplantation under the kidney capsule of NOD/SCID (CD45.1) mice. Grafts were dissected out after 4 weeks and cells dissociated for antibody staining and flow cytometry. Live singlets were gated, and CD11b, CD11c and F4/80 staining was used to identify myeloid cell subsets. Staining for CD3 and CD19 expression on the gated CD11b^-^CD11c^-^ population was used to identify lymphoid cells. Three individual grafts transplanted under the kidney capsule of a single mouse were analysed, and cell composition compared with splenic leukocytes from adult C57BL/6J and NOD/SCID mice.
(PDF)

S1 Table Summary of individual grafting experiments. The 5G3 and 3B5 stromal cells were harvested and prepared for grafting by either overnight cultures on a collagen sponge, or by mixing with Matrigel ahead of surgical implantation under the kidney capsule of NOD/SCID mice.
(PDF)
