# Supplementary figures and images for: Transplanted spleen stromal cells with osteogenic potential support ectopic myelopoiesis
Source: PLoS One. 2019 Oct 4;14(10):e0223416. doi: 10.1371/journal.pone.0223416 (PMC6777786; doi:10.1371/journal.pone.0223416)

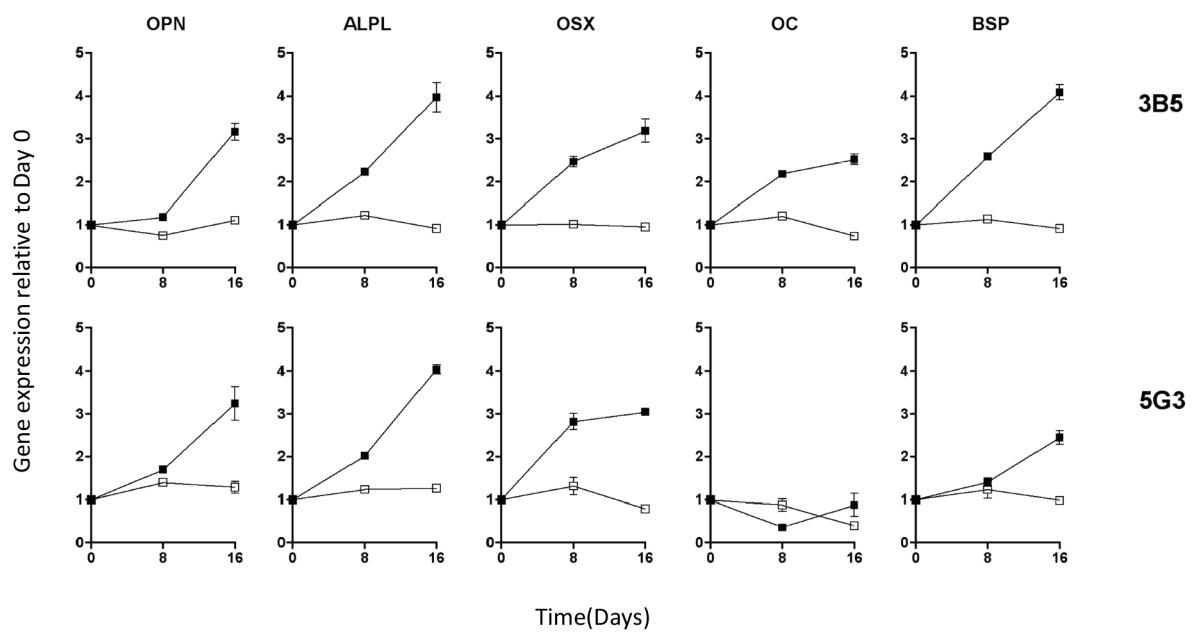

Supplement: S2 Fig — A repeat experiment was performed to assess expression of genes reflecting osteogenesis upon culture of 5G3 and 3B5 stroma under mineralization conditions. Quantitative RT-PCR was used to measure change in gene expression over time in culture for genes encoding alkaline phosphatase (ALPL), osterix (OSX), osteocalcin (OC), bone sialoprotein (BSP) and osteopontin (OPN) by 5G3 and 3B5 stromal cells induced to undergo osteogenic differentiation (closed symbols). 5G3 and 3B5 grown under normal culture conditions served as control cells (open symbols). Data are shown as fold change in gene expression at 8-day intervals relative to Day 0 gene expression. Data points represent mean ° SE of three experimental replicates. (PDF) [file pone.0223416.s002.pdf]

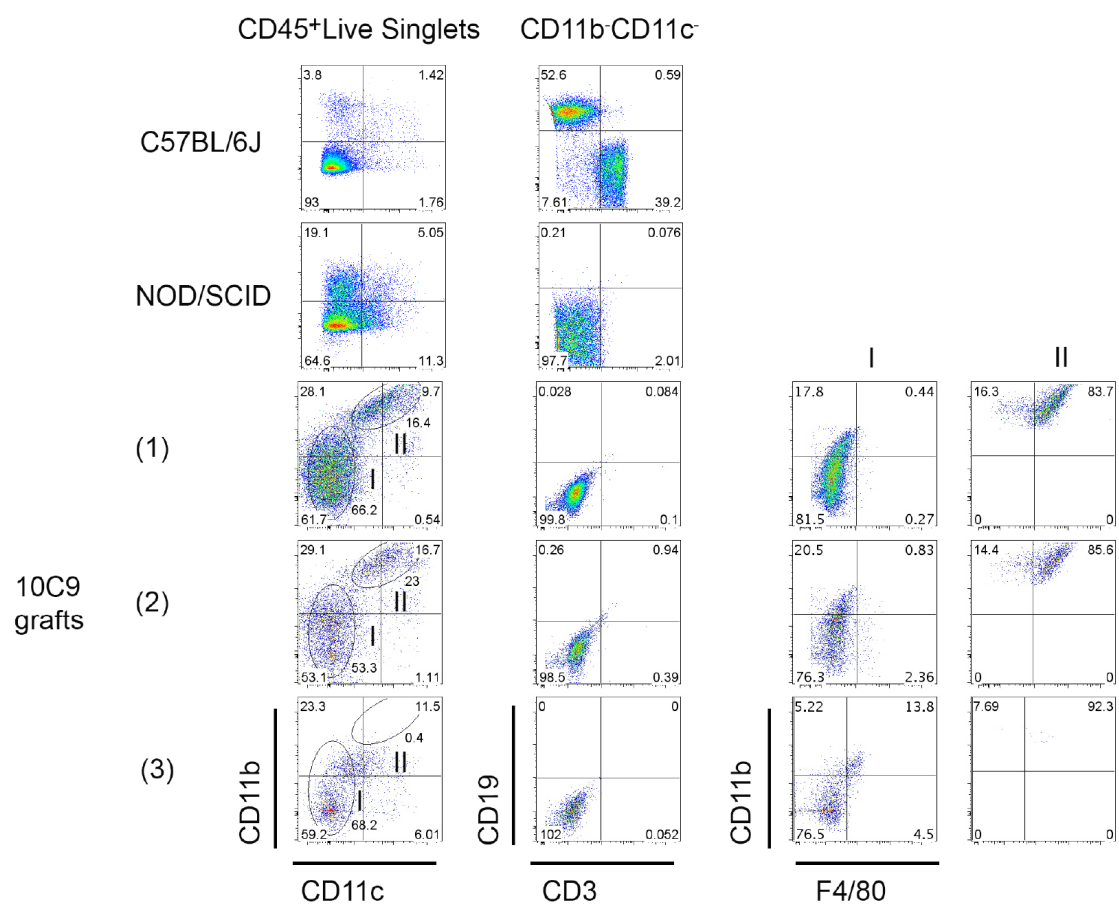

Supplement: S3 Fig — 10C9 stromal cells were grown on a collagen sponge ahead of transplantation under the kidney capsule of NOD/SCID (CD45.1) mice. Grafts were dissected out after 4 weeks and cells dissociated for antibody staining and flow cytometry. Live singlets were gated and staining for CD11b, CD11c and F4/80 used to identify myeloid subsets. Staining for CD3 and CD19 expression on the gated CD11b-CD11c- population was used to identify lymphoid cells. Three separate grafts from individual mice were analysed and cell composition compared with spleen leukocytes from adult C57BL/6J and NOD/SCID mice. (PDF) [file pone.0223416.s003.pdf]

CD11b-CD11c-

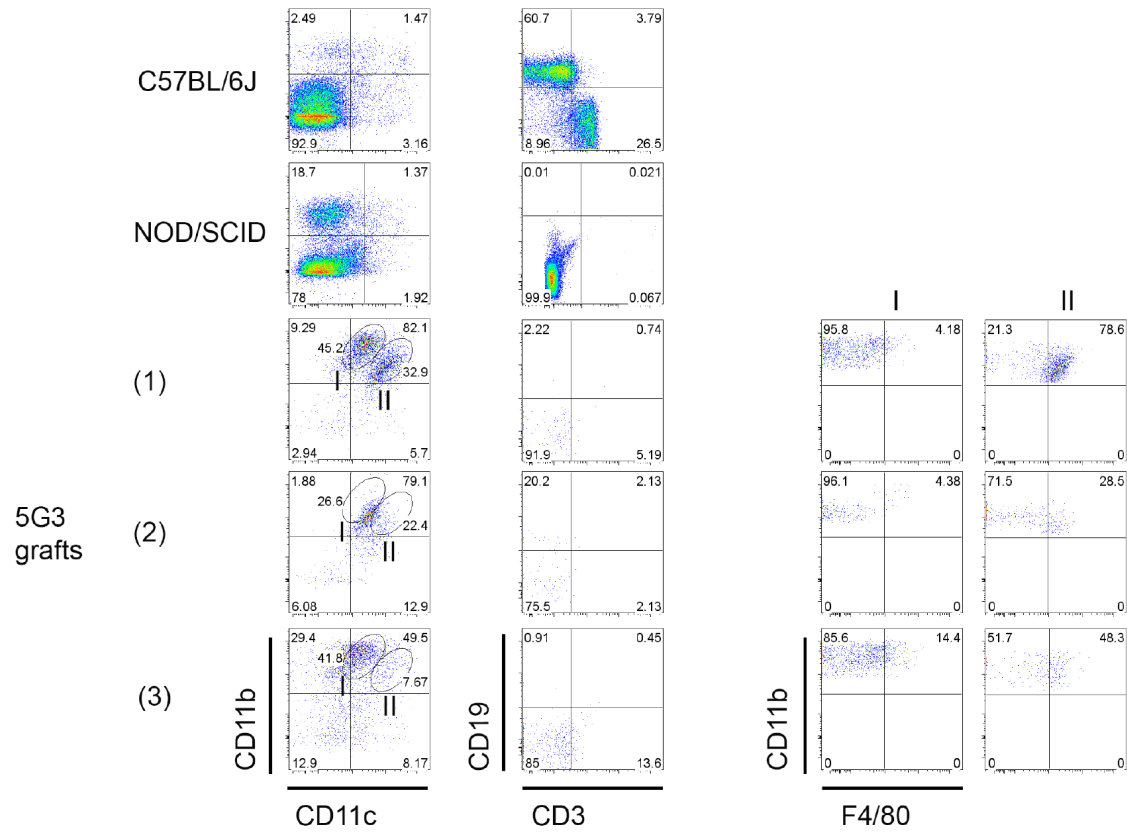

Supplement: S4 Fig — 5G3 stromal cells were grown on a collagen sponge ahead of transplantation under the kidney capsule of NOD/SCID (CD45.1) mice. Grafts were dissected out after 4 weeks and cells dissociated for antibody staining and flow cytometry. Live singlets were gated, and CD11b, CD11c and F4/80 staining was used to identify myeloid cell subsets. Staining for CD3 and CD19 expression on the gated CD11b-CD11c- population was used to identify lymphoid cells. Three individual grafts transplanted under the kidney capsule of a single mouse were analysed, and cell composition compared with splenic leukocytes from adult C57BL/6J and NOD/SCID mice. (PDF) [file pone.0223416.s004.pdf]
