# Supplementary material for: Transplanted spleen stromal cells with osteogenic potential support ectopic myelopoiesis
Source: PLoS One. 2019 Oct 4;14(10):e0223416. doi: 10.1371/journal.pone.0223416 (PMC6777786; doi:10.1371/journal.pone.0223416)
Supplement: S1 Table — The 5G3 and 3B5 stromal cells were harvested and prepared for grafting by either overnight cultures on a collagen sponge, or by mixing with Matrigel ahead of surgical implantation under the kidney capsule of NOD/SCID mice. (PDF) [file pone.0223416.s005.pdf]

S1 Table. Summary of individual grafting experiments.

Stroma	No. of cells/graft	Matrix type	No. of grafted mice	Graft analysis (weeks)	No. of successful grafts	% success rate
3B5	10^6	Collagen sponge	19	4	13	68
	10^6	Matrigel	2	4	2	100
	$5x10^4$: $5x10^5$ [b]	Matrigel	2	2	1[1]	50
5G3	10^6	Collagen sponge	24 (4[a])	4	3[c]	12.5
	10^6	Matrigel	4	4	0	0
	10^6	Matrigel	3	3	0	0
	$5x10^4$: $5x10^5$ [b]	Matrigel	2	1 : 2	0	0
10C9	$1x10^6$	Matrigel	5	4	3	60
STX3	$1x10^6$	Matrigel	5	4	0	0
7G10	$1x10^6$	Matrigel	5	4	0	0

The 5G3 and 3B5 stromal cells were harvested and prepared for grafting by either overnight culture on a collagen sponge, or by mixing with Matrigel ahead of surgical implantation under the kidney capsule of NOD/SCID mice.

[a] Some mice were splenectomised ahead of grafting.

[b] Some mice were given 2 distinct grafts of different size on the same kidney.

[c] Three grafts on the same animal.
