# Supplementary material for: Transplanted spleen stromal cells with osteogenic potential support ectopic myelopoiesis
Source: PLoS One. 2019 Oct 4;14(10):e0223416. doi: 10.1371/journal.pone.0223416 (PMC6777786; doi:10.1371/journal.pone.0223416)
Supplement: S1 Fig — Data mining was applied to Affymetrix datasets collected for stromal cells. Log2 average signal value was plotted as a heat map, and overlaid dashed blue line represents signal changes around mean). Sets of genes reflecting chemokine, and cytokine pathways represent genes utilized by SASBioscience for their PCR arrays. (PDF) [file pone.0223416.s001.pdf]

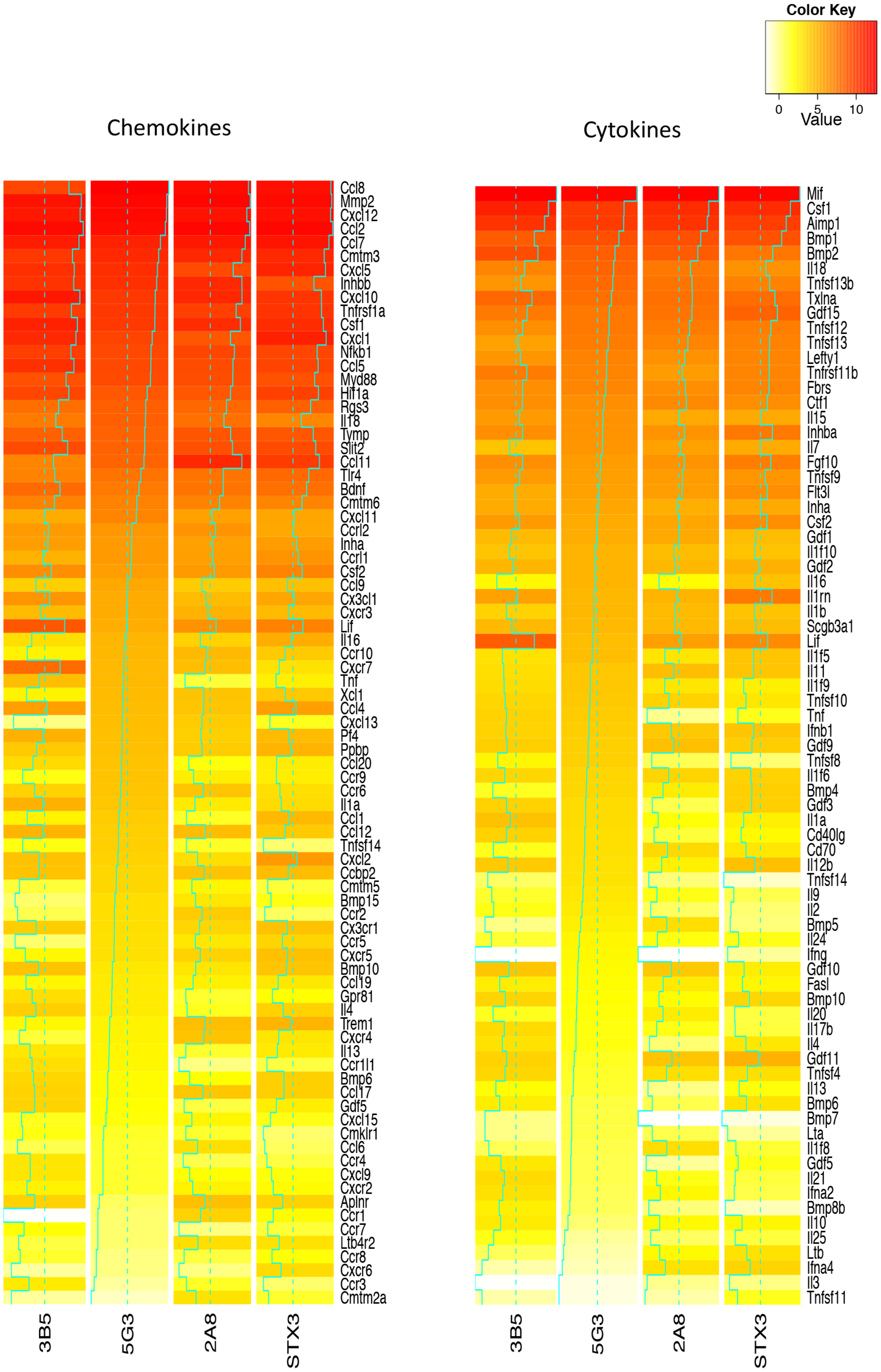

Color Key
0
5
10
Value
Chemokines
Ccl8
Mmp2
Cxcl12
Ccl2
Ccl7
Cmtm3
Cxcl5
Inhbb
Cxcl10
Tnfrsf1a
Csf1
Cxcl1
Nfkb1
Ccl5
Myd88
Hif1a
Rgs3
Il18
Tymp
Slit2
Ccl11
Tlr4
Bdnf
Cmtm6
Cxcl11
Ccrl2
Inha
Ccrl1
Csf2
Ccl9
Cx3cl1
Cxcr3
Lif
Il16
Ccr10
Cxcr7
Tnf
Xcl1
Ccl4
Cxcl13
Pf4
Ppbp
Ccl20
Ccr9
Ccr6
Il1a
Ccl1
Ccl12
Tnfsf14
Cxcl2
Ccbp2
Cmtm5
Bmp15
Ccr2
Cx3cr1
Ccr5
Cxcr5
Bmp10
Ccl19
Gpr81
Il4
Trem1
Cxcr4
Il13
Ccr1l1
Bmp6
Ccl17
Gdf5
Cxcl15
Cmklr1
Ccl6
Ccr4
Cxcl9
Cxcr2
Aplnr
Ccr1
Ccr7
Ltb4r2
Ccr8
Cxcr6
Ccr3
Cmtm2a
3B5
5G3
2A8
STX3
Cytokines
Mif
Csf1
Aimp1
Bmp1
Bmp2
Il18
Tnfsf13b
Txlna
Gdf15
Tnfsf12
Tnfsf13
Lefty1
Tnfrsf11b
Fbrs
Ctf1
Il15
Inhba
Il7
Fgf10
Tnfsf9
Flt3l
Inha
Csf2
Gdf1
Il1f10
Gdf2
Il16
Il1rn
Il1b
Scgb3a1
Lif
Il1f5
Il11
Il1f9
Tnfsf10
Tnf
Ifnb1
Gdf9
Tnfsf8
Il1f6
Bmp4
Gdf3
Il1a
Cd40lg
Cd70
Il12b
Tnfsf14
Il9
Il2
Bmp5
Il24
Ifng
Gdf10
Fasl
Bmp10
Il20
Il17b
Il4
Gdf11
Tnfsf4
Il13
Bmp6
Bmp7
Lta
Il1f8
Gdf5
Il21
Ifna2
Bmp8b
Il10
Il25
Ltb
Ifna4
Il3
Tnfsf11
3B5
5G3
2A8
STX3
